# Cross reactivity of neutralizing antibodies to the encephalitic California Serogroup orthobunyaviruses varies by virus and genetic relatedness

**DOI:** 10.1038/s41598-021-95757-2

**Published:** 2021-08-12

**Authors:** Alyssa B. Evans, Karin E. Peterson

**Affiliations:** Laboratory of Persistent Viral Diseases, Rocky Mountain Laboratories, National Institute of Allergy and Infectious Diseases, National Institutes of Health, Hamilton, MT 59840 USA

**Keywords:** Infection, Humoral immunity, Virus-host interactions

## Abstract

The California Serogroup (CSG) of Orthobunyaviruses comprises several viruses capable of causing neuroinvasive disease in humans, including La Crosse (LACV), Snowshoe Hare (SSHV), Tahyna (TAHV), Jamestown Canyon (JCV), and Inkoo (INKV) viruses. Diagnosis of specific CSG viruses is complicated by the high degree of antibody cross-reactivity between them, with laboratory standards requiring a fourfold higher titer of neutralizating antibody (NAb) activity to positively identify the etiologic virus. To help elucidate NAb relationships between neuroinvasive CSG viruses, we directly compared the cross-reactivity of NAb between LACV, SSHV, TAHV, JCV, and INKV. Mice were inoculated with individual viruses and the NAb activity of plasma samples was compared by plaque reduction neutralization tests against all five viruses. Overall, the results from these studies show that the CSG viruses induced high levels of NAb against the inoculum virus, and differing amounts of cross-reactive NAb against heterologous viruses. LACV, SSHV, and INKV elicited the highest amount of cross-reactive NAb. Interestingly, a fourfold difference in NAb titer between the inoculum virus and the other CSG viruses was not always observed. Thus, NAb titers, which are the gold-standard for diagnosing the etiologic agent for viral encephalitis, may not clearly differentiate between different CSG viruses.

## Introduction

The California Serogroup (CSG) is a group of at least 18 antigenically and genetically related mosquito-borne viruses in the Family *Peribunyaviridae* of the Genus *Orthobunyavirus*^1^. Several viruses within the CSG have been reported to cause neuroinvasive disease in humans, including La Crosse virus (LACV), Snowshoe hare virus (SSHV), Tahyna virus (TAHV), Jamestown Canyon virus (JCV), and Inkoo virus (INKV)^1–5^. These five viruses are found throughout most of the world with discrete, but sometimes overlapping, geographic distributions. LACV, SSHV, and JCV are all found in the USA, with SSHV and JCV extending into Canada^1^. TAHV is widespread throughout Europe, Asia, and Africa, and INKV is primarily found in Scandinavia, but overlaps with TAHV in Russia^1^. Additionally, there is overlap between the mosquito vectors and vertebrate hosts utilized by these viruses^1^.

Serogroup classification of viruses has traditionally been a way to group similar viruses based on the cross-reactivity of the antibodies they elicit. More recently, genetic classification of viruses has become the standard as sequencing techniques become cheaper and easier. While genetic classification gives a better understanding of the relatedness of viruses, serological relationships are still important to understand because of their implications for vaccine development and diagnostics. This is especially true for the neuroinvasive CSG viruses, as a diagnosis usually relies on serology. The virus is typically not detectable in blood or cerebrospinal fluid by the time patients present with neurological symptoms, ruling out a PCR-based approach as a diagnostic tool in most cases. A better understanding of the relationships of cross-reactive antibody between the neuroinvasive CSG viruses is critical for improving diagnosis of these viruses.

**Figure 1:**
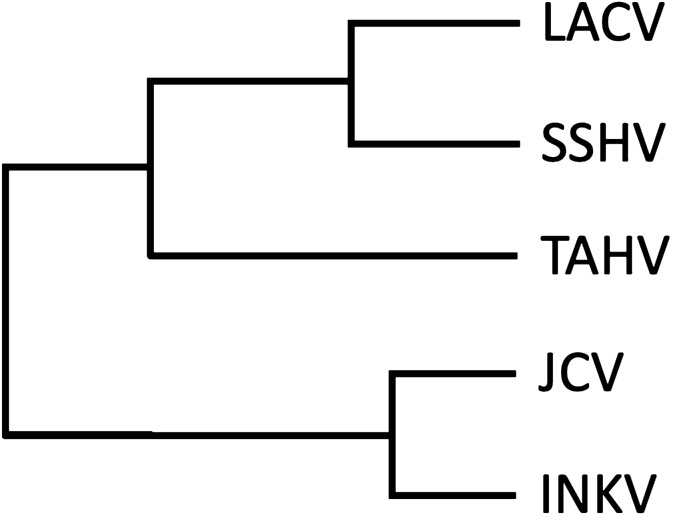
Schematic of genetic relationships between the CSG viruses used in this study. Figure was constructed in Microsoft Office 16 Powerpoint.

The genetic relationships of LACV, SSHV, TAHV, JCV, and INKV have previously been evaluated in several phylogenetic studies, with similar relationships found across all segments. As summarized schematically in Fig. 1, JCV and INKV are most closely related to each other and are grouped in the Melao (MELV) complex, LACV and SSHV are most closely related to each other and are grouped in the California Encephalitis (CEV) complex^6–8^. TAHV is also in the CEV complex and is more closely related to LACV and SSHV than to JCV and INKV^7,8^. Serological relationships between these viruses have been inferred from a variety of antibody and haemagglutination assays, using different strain combinations. Historically, LACV, TAHV, SSHV, and INKV were grouped together in the California Encephalitis Complex, and JCV in the Melao Complex^9–11^. However, the early studies upon which these classifications were made relied on crude serological techniques, often compared a limited number of CSG viruses, and did not have sequence information to verify the viruses used for comparisons. While these studies are informative, there has not been a study to directly compare the differences in cross-reactive antibodies induced by the clinically-relevant neuroinvasive CSG viruses.

To evaluate the cross-reactivity of antibodies elicited by LACV, SSHV, TAHV, JCV, and INKV in as controlled of a system as possible, we inoculated adult mice that are resistant to CSG virus-induced neuroinvasive disease intraperitoneally (IP) with each of these five viruses. Plasma was taken from the mice four weeks after inoculation and evaluated for neutralizing antibody (NAb) against the inoculum (homologous) virus and the other four (heterologous) CSG viruses by plaque reduction neutralization test (PRNT) to compare NAb titers and cross-reactivity. While mice and humans do have differences in specific antibody subtypes and mechanisms of development^12^, the general function of antibodies and their role in neutralization is similar between mice and humans. Additionally, we have previously shown that pathogensis of these five CSG viruses in mice is similar to what is observed in humans^13^. Therefore, determining the level of cross-reactivity of NAb to the CSG viruses in mice will provide insights into the human NAb response to CSG viruses.

The orthobunyavirus spike is comprised of three heterodimers of the two envelope proteins, Gn and Gc that are arranged in a tripod-like structure on the virion surface^14^. This complex envelope protein arrangement has made it difficult to determine specific neutralizing epitopes for the CSG viruses. However, the Gc protein, and particularly the head domain of the Gc protein, has been indicated as the primary target for NAbs^15,16^. We therefore sequenced our stock viruses to determine the exact genetic relatedness between the viruses used in this study. We then compared the cross-reactivity of NAbs between viruses to a comparison of genetic identity of the two envelope proteins, Gc and Gn, with a particular focus on the Gc head domain.

## Results

### CSG viruses induced high levels of homologous neutralizing antibody

**Figure 2:**
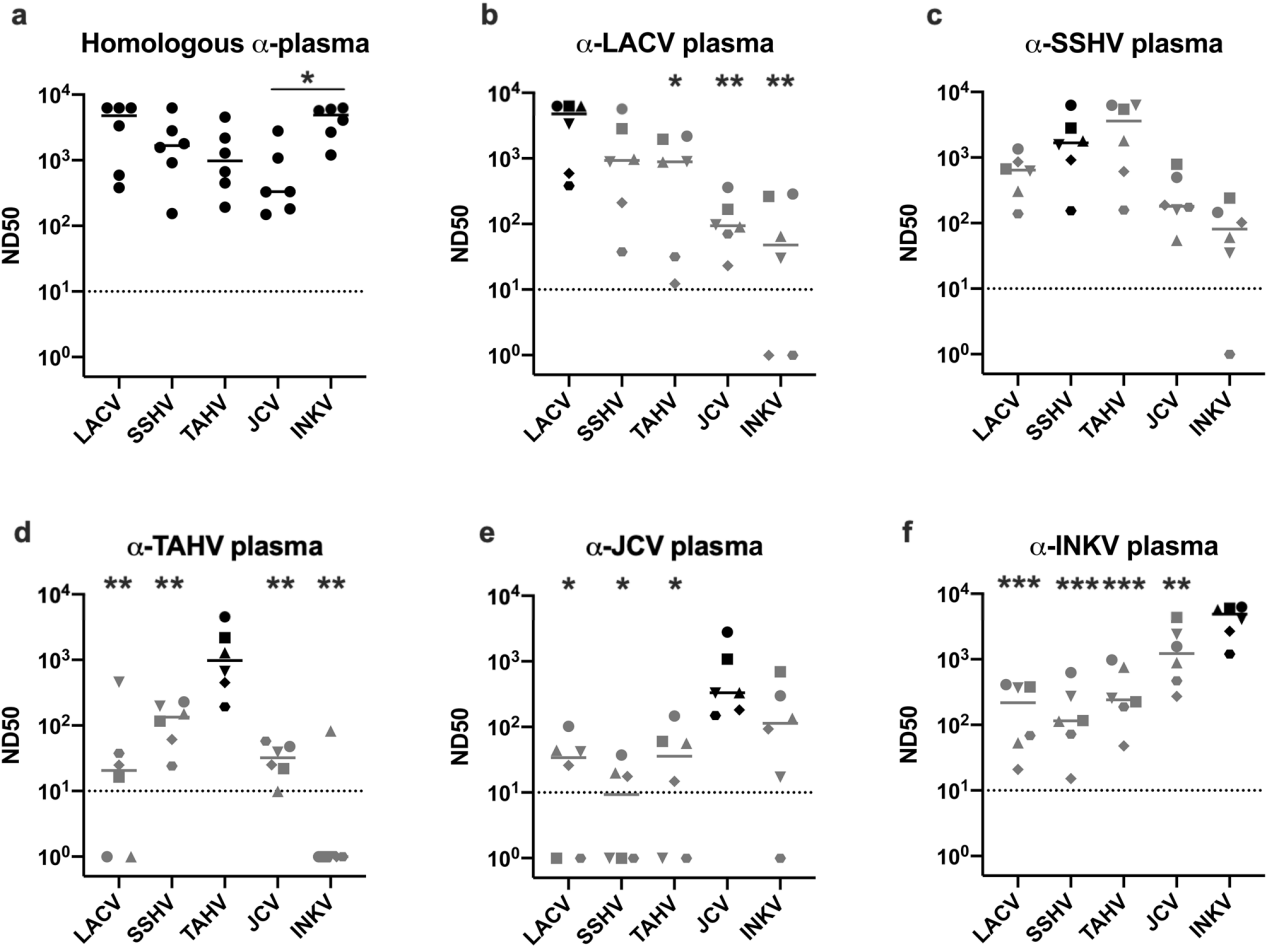
Homologous and heterologous neutralization of CSG viruses. (**a**) Comparison of ND50 values for homologous viruses. (**b**–**f**) Comparison of ND50 values for each anti-plasma sample set against the homologous (inoculum) virus (black) and the other four heterologous viruses (gray). Within graphs (**b**–**f**), individual symbols correspond to the same plasma sample. Data analyzed via One-way ANOVA with Dunnett’s multiple comparison post-test for all sample combinations (**a**), or compared with homologous ND50 values only (**b**–**f**) in GraphPad Prism 8. Significance values are designated as *p = 0.05–0.01, **p = 0.009–0.001, ***p ≤ 0.0009. Dotted lines represent ND50 = 10, which was the lowest dilution used in the assay.

To evaluate the neutralizing antibody (NAb) response to the CSG viruses, we inoculated adult mice intraperitoneally (IP) with 10^5^ PFU of LACV, SSHV, TAHV, JCV, or INKV. At 29–30 days post inoculation (dpi), mice were euthanized and blood plasma collected. None of the mice developed clinical disease. PRNT of plasma samples were performed against the homologous virus in Vero cells. The dilution that neutralized 50% of the input virus (ND50) was calculated to evaluate the level of NAb each virus elicited in the mice. All five of the CSG viruses induced high levels of homologous NAb in all mice (Fig. 2A). Within each virus there was a range of ND50 values, with LACV and INKV inducing the highest levels of homologous NAb, then SSHV, TAHV, and JCV (Fig. 2A, Table 1). Comparison of homologous NAb titers revealed similar ND50 levels for all of the viruses, with the only significant difference between JCV and INKV (p = 0.044). One caveat to these findings is that LACV and INKV had multiple samples whose ND50 was at the limit of the highest dilution we tested (1:6250), so the absolute number for those samples might be higher. However, the results still reflect the trend of the NAb levels.

**Table 1:** Median ND50.

	Virus				
	LACV	SSHV	TAHV	JCV	INKV
**ɑ-plasma**					
LACV	*4795.0*	932.2	887.3	93.8	47.6
SSHV	645.1	*1677.0*	3621.0	180.7	81.3
TAHV	20.7	133.7	*977.4*	32.1	1.0
JCV	33.9	9.3	35.7	*331.3*	114.1
INKV	217.9	115.2	240.6	1225.0	*4895.0*

Italicized values indicate homologous virus median ND50.

We next evaluated if the differences in homologous neutralizing antibody response could be due to sex differences. While we did not specifically design this study to evaluate sex differences, we did use a mix of male and female mice. We first evaluated if there was a difference between homologous virus ND50 values between male and female mice by combining the homologous ND50s for all viruses (males = 17, females = 13), but there was no significant difference via t-test (p = 0.527). We next compared homologous ND50 values between males and females within each virus (except LACV, which only had one female), where SSHV and TAHV had three males and three females each, JCV had four males and two females, and INKV had two males and four females. No significant differences were found between males and females for any of the viruses (p ≥ 0.196). While we cannot say conclusively that there is not a sex difference due to the small sample sizes, we did not observe a difference in neutralizing antibody response to the CSG viruses between male and female mice.

### CSG viruses induced differing levels of cross-reacting NAb

We next evaluated the amount of cross-reaction of NAbs between the five CSG viruses by performing PRNTs of plasma samples against the four heterologous CSG viruses. The α-LACV, α-TAHV, α-JCV, and α-INKV plasma samples all neutralized homologous virus better than the heterologous viruses, but with virus and sample variation (Fig. 2B,D,E,F, Table 1). α-LACV plasma induced high levels of cross-reactive NAb against the four heterologous viruses (Fig. 2B). The highest level of cross-reactive NAb was against SSHV, then TAHV, with a substantial drop-off against JCV and INKV (Fig. 2B, Table 1). Only two α-LACV samples failed to reach an ND50 ≥ 10, the lowest dilution tested, for all viruses. Both of the samples were unable to inhibit INKV (Fig. 2B), one of the viruses most distant from LACV (Fig. 1). The median ND50 value was higher for α-LACV plasma against homologous LACV than against its closest relative, SSHV, and there was more variability against SSHV, but no statistical difference in their ND50s (Fig. 2B, Tables 1, 2). In comparison, there was a statistically significant difference between α-LACV plasma homologous virus ND50 versus the other three heterologous viruses’ ND50s (Fig. 2B, Tables 1, 2).

**Table 2:**
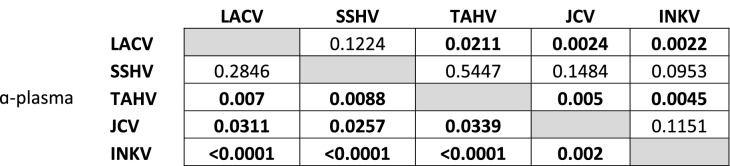
P values comparing ND50s of homologous vs. heterologous viruses.

P value was determined by One-way ANOVA with a Dunnett's multiple comparison post-test in Prism.

Bold = significant.

Shaded cells indicates homologous virus.

Surprisingly, α-SSHV plasma neutralized TAHV better than homologous SSHV, but also had high levels of cross-reactivity against LACV, JCV, and INKV (Fig. 2C, Table 1). However, none of the heterologous virus ND50s for the α-SSHV plasma were significantly different from the SSHV ND50 via One-way ANOVA with Dunnett's multiple comparison analysis (Table 2).

α-TAHV and α-JCV plasma had poorer cross-neutralizing antibodies compared to α-LACV plasma. α-TAHV plasma had significantly higher ND50s against TAHV than all four heterologous viruses. The highest level of cross-neutralizing antibody was against SSHV, followed by JCV then LACV (Fig. 2D, Tables 1, 2). Only one of the six α-TAHV plasma samples had detectable NAb against INKV (Fig. 2D). The α-JCV plasma had significantly higher homologous virus ND50 than LACV, SSHV, and TAHV ND50s, but was not significantly different from its closest relative, INKV (Fig. 2E, Table 2). At least one α-JCV plasma sample failed to elicit NAb titers ≥ 10 for each of the four heterologous viruses (Fig. 2E). α-INKV plasma had a high level of cross-reactivity against the four heterologous viruses. Every α-INKV plasma sample reached ND50 values ≥ 10 against every virus tested, with the highest level of cross-reactive NAb against JCV (Fig. 2F). However, all heterologous viruses had significantly lower ND50s than INKV (Table 2).

Overall, α-LACV, α-SSHV, and α-INKV plasma induced the highest homologous and cross-reactive NAb titers amongst the tested CSG viruses, while α-TAHV and α-JCV plasma had the lowest cross-reactivity. For almost all plasma samples, homologous virus was neutralized better than heterologous viruses. These findings show that the CSG viruses elicit differing levels of cross-reactive antibody, depending on the α-plasma and virus tested. Generally, neutralizing antibody titers correlated with genetic relatedness, with the closest genetically related heterologous virus having higher ND50 values than the more distantly related heterologous viruses (Figs. 1, 2A,E,F, Table 1). Surprisingly, the α-SSHV plasma actually had higher neutralizing activity against TAHV than SSHV, but not against LACV, which SSHV is more closely related to. This indicates that the level cross-reactivity is not necessarily directly correlated with genetic relatedness.

### CSG viruses were often not discriminated by a fourfold difference in NAb titer

A fourfold difference in NAb titer is often used as a diagnostic cutoff for determining the etiologic agent of a CSG virus infection^17,18^. Therefore, we next looked at the fold difference between homologous virus and each heterologous virus for every plasma sample to determine how often we could discriminate the inoculum virus from our known samples with a fourfold difference. Overall, the ability to discriminate CSG viruses by a fourfold difference in NAb titer differed between CSG viruses, but this cutoff was most often not reached between genetically more closely related viruses (Tables 3, 4, 5, 6, 7). LACV was discriminated from JCV and INKV in all six of the α-LACV plasma samples, but in only three samples against TAHV, and two samples against SSHV (Table 3). The inability to dismcrimate by ≥ fourfold correlated with LACV being most closely related to SSHV, then TAHV, then JCV and INKV.

**Table 3:** ND50 fold difference of ɑ-LACV plasma between heterologous viruses vs. homologous LACV.

	SSHV	TAHV	JCV	INKV
ɑ-LACV-1*	*2.2*	*3.2*	37.4	23.7
ɑ-LACV-2	*2.8*	48.4	25.6	591.6
ɑ-LACV-3	*3.7*	*3.7*	34.2	110.8
ɑ-LACV-4*	6.4	7.1	69.5	96.1
ɑ-LACV-5	10.2	12.1	5.5	383.5
ɑ-LACV-6*	*1.1*	*2.9*	17.4	21.8
Average	4.4	12.9	31.6	204.6

*Homologous ND50 > 6250, but 6250 highest dilution tested and value used.

Italics = < fourfold difference.

**Table 4:** ND50 fold difference of ɑ-SSHV plasma between heterologous viruses vs. homologous SSHV

	LACV	TAHV	JCV	INKV
ɑ-SSHV-1	*2.5*	**− *4.0***	9.8	44.7
ɑ-SSHV-2	4.2	**− *1.9***	*3.6*	11.8
ɑ-SSHV-3	*1.1*	*1.5*	4.9	9.0
ɑ-SSHV-4	*1.1*	*1.0*	**− *1.1***	154.2
ɑ-SSHV-5	5.9	*1.0*	32.7	29.5
ɑ-SSHV-6*	4.6	*1.0*	12.6	42.8
Average	*3.2*	*0.9*	10.7	48.7

*Homologous ND50 > 6250, but 6250 highest dilution tested and value used.

Italics = < fourfold difference.

Bold = higher heterologous ND50.

**Table 5:** ND50 fold difference of ɑ-TAHV plasma between heterologous viruses vs. homologous TAHV.

	LACV	SSHV	JCV	INKV
ɑ-TAHV-1	5.1	8.0	*3.3*	192.6
ɑ-TAHV-2	18.1	7.4	18.0	451.7
ɑ-TAHV-3	*1.5*	*3.4*	17.1	668.7
ɑ-TAHV-4	4533.0	19.8	94.8	4533.0
ɑ-TAHV-5	132.6	18.6	99.1	2173.0
ɑ-TAHV-6	1286.0	8.5	130.6	15.6
Average	996.0	10.9	60.5	1339.1

Italics = < fourfold difference.

**Table 6:** ND50 fold difference of ɑ-JCV plasma between heterologous viruses vs. homologous JCV.

	LACV	SSHV	TAHV	INKV
ɑ-JCV-1	149.2	149.2	149.2	149.2
ɑ-JCV-2	7.0	10.3	12.2	*1.9*
ɑ-JCV-3	7.5	16.7	5.9	*2.5*
ɑ-JCV-4	27.5	74.7	19.1	9.3
ɑ-JCV-5	7.9	330.0	330.0	19.2
ɑ-JCV-6	1083.0	1083.0	18.0	*1.6*
Average	213.7	277.3	89.1	30.6

Italics = < fourfold difference.

**Table 7:** ND50 fold difference of ɑ-INKV plasma between heterologous viruses vs. homologous INKV

	LACV	SSHV	TAHV	JCV
ɑ-INKV-1	11.1	14.9	15.9	*1.7*
ɑ-INKV-2	15.8	51.5	26.7	*1.4*
ɑ-INKV-3*	15.1	10.0	6.4	4.0
ɑ-INKV-4	107.3	50.4	7.5	6.5
ɑ-INKV-5	17.5	16.7	6.4	*2.5*
ɑ-INKV-6	127.7	176.0	55.8	9.8
Average	49.1	53.2	19.8	4.3

*Homologous ND50 > 6250, but 6250 highest dilution tested and value used.

Italics = < fourfold difference.

SSHV was the least often discriminated by a ≥ fourfold difference (Table 4). All six α-SSHV samples had a greater than fourfold difference against INKV. Against JCV, four of six samples had a greater than fourfold difference, but one sample actually had a higher titer against JCV than SSHV (Table 4). Against SSHV’s closest relative, LACV, SSHV was discriminated by a ≥ fourfold difference in only three of six samples. None of the six α-SSHV plasma samples had a ≥ fourfold difference between SSHV and TAHV that discriminated SSHV as the inoculum virus, and in fact one sample had a fourfold higher ND50 against TAHV than SSHV (Table 4). In contrast, α-TAHV samples were discriminated by ≥ fourfold difference in all six samples against INKV, and in five of six samples against LACV, SSHV, and JCV (Table 5). For the α-JCV and α-INKV plasma samples, the inoculum virus was discriminated by ≥ fourfold in all six samples against LACV, SSHV, and TAHV (Tables 6, 7). Against the more closely related JCV and INKV, the α-JCV and α-INKV plasma only discriminated the inoculum virus by a ≥ fourfold difference in three samples (Tables 6, 7).

Overall, plasma samples were not consistently discriminated by a ≥ fourfold difference in NAb titer between the inoculum virus and heterologous CSG viruses. However, the inoculum virus was always neutralized with the highest titer for all viruses, except SSHV. α-SSHV plasma had equal or lower homologous NAb titers compared to TAHV in five of the six samples, and against JCV in only one sample, indicating that SSHV induces a higher level of cross-reactive NAb compared to the other CSG viruses.

### Genetic and antigenic relatedness of the CSG viruses did not always correlate

Genetic relatedness of the CSG viruses has been evaluated in a variety of phylogenetic studies, with a consensus for the relationships between LACV, SSHV, TAHV, JCV, and INKV determined using a variety of strains that differed between studies^6–8^. Therefore, to determine the exact genetic relatedness of the viruses used in our study, we sequenced our virus stocks. The sequence identity of these specific stocks followed previously identified relationships, with JCV and INKV being the most closely related to each other, then SSHV and LACV, with TAHV being more closely related to SSHV and LACV than to JCV and INKV (Table 8). Overall, Gn was more similar across the CSG viruses than Gc, with amino acid identities for Gn ranging between 98% (JCV and INKV) to 88.1% (JCV and TAHV), whereas Gc amino acid identities ranged from 92.8% (JCV and INKV) to 68.8% (JCV and LACV) (Table 8).

**Table 8:**
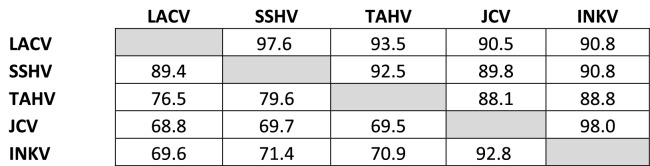
Amino acid % identities for Gn (above shaded cells) and Gc (below shaded cells).

**Table 9:**
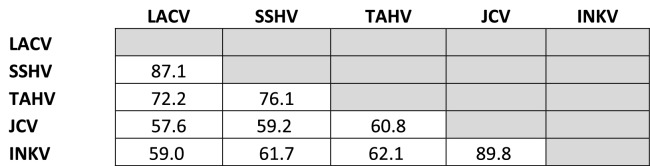
Amino Acid identities for the Gc Head domain.

The amino acid % identity decreased when looking specifically at the Gc head domain, which has been implicated as the main target for host neutralizing antibodies^15^. For the Gc head domain, amino acid % identity ranged from 89.8% (JCV and INKV) to 57.6% (JCV and LACV) (Table 9). We calculated the correlation of percent identities within the Gc head domain with heterologous ND50s for plasma from each inoculum virus. For all α-plasma except α-SSHV, there was a strong positive correlation of Gc head domain % identities with heterologous ND50 (α-LACV r^2^ = 0.995, p = 0.0025; α-SSHV r^2^ = 0.148, p = 0.6153; α-TAHV r^2^ = 0.943, p = 0.0289; α-JCV r^2^ = 0.976, p = 0.0121; α-INKV r^2^ = 0.987, p = 0.0066).

**Figure 3:**
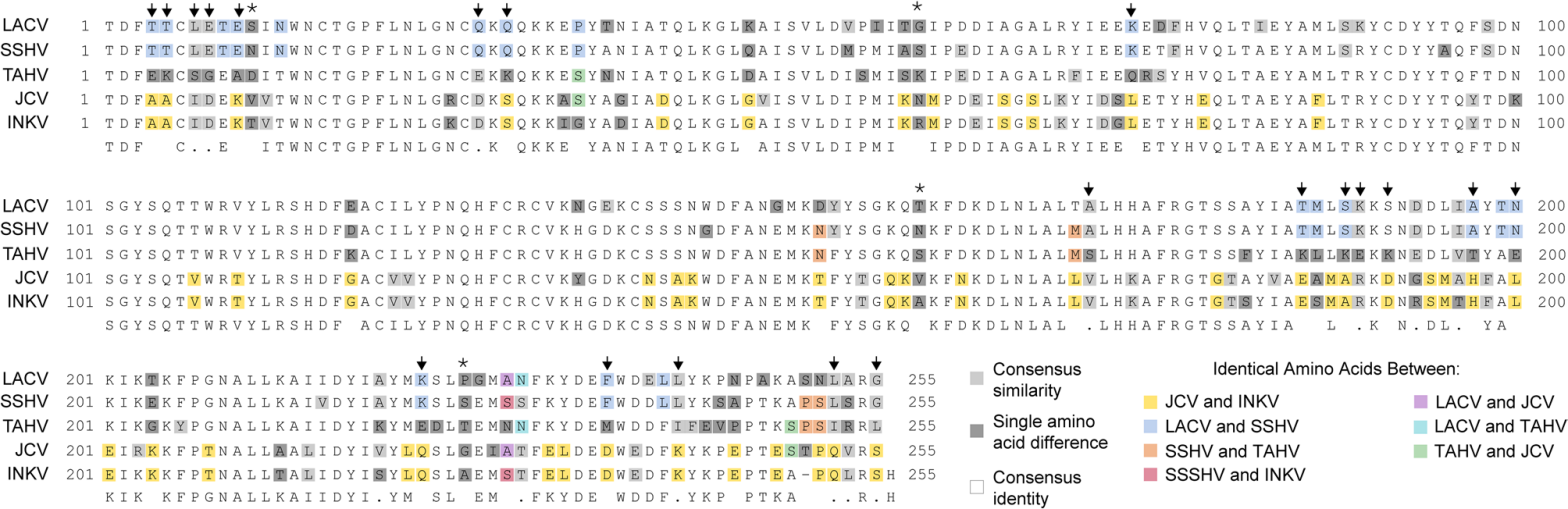
Gc Head Domain alignment. No shading indicates consensus amino acid identities across the five sequences, light gray shading indicates consensus similarities across the five sequences, dark gray shading indicates differences in individual virus amino acid, and color shading indicates shared amino acids between two viruses (see legend). Arrows point out sites where LACV & SSHV share, JCV & INKV share, and TAHV has a different amino acid. Asterisks show sites where all five viruses have a different amino acid. Sequence alignments were performed in MacVector version 17.5.4, and the figure constructed in Adobe Photoshop.

A sequence alignment of the Gc head domain between the five CSG viruses showed variability within this region (Fig. 3). As expected based on the % identity comparisons, in regions without a consensus amino acid between the five viruses, often LACV and SSHV shared amino acids (Fig. 3, blue shading), JCV and INKV shared amino acids (Fig. 3, yellow shading), and TAHV had a different amino acid from the other four (Fig. 3, arrows). There were only four sites where each virus had a different amino acid (Fig. 3, asterisks). However, there were a few residues where more distantly related viruses shared amino acids different from the others. There were two positions where TAHV and JCV shared amino acids (Fig. 3, green shading), one position where LACV and JCV shared amino acids (Fig. 3, purple shading), one position where SSHV and INKV shared amino acids (Fig. 3, red shading), and one position where LACV and TAHV shared amino acids (Fig. 3, aqua shading). Interestingly, given the high level of cross reactive NAb SSHV elicited to TAHV, there were four sites where TAHV and SSHV shared amino acids (Fig. 3, orange shading) different from the other three viruses, although it is unknown if these sites mediate that cross-reactivity. Overall, although there were some differences in amino acids within the Gc head domain, there was not an obvious region that correlated with the differences in cross-neutralization between these CSG viruses.

## Discussion

The CSG viruses have been well established to be a closely related group of viruses both genetically and antigenically. Previous studies have shown varying degrees of antibody cross-reactivity between different members of the CSG, often using patient or vertebrate host sera without knowing the origin of the viral infection, let alone the specific strain of virus. In the current study, all CSG viruses induced high levels of homologous NAb in mice, and for all inoculum viruses except SSHV, the homologous virus was neutralized better than the heterologous viruses (Fig. 2, Table 1). The level of NAb to heterologous viruses varied by inoculum virus and often, but not always, correlated with genetic relatedness. The notable exceptions to this were the α-SSHV and α-TAHV plasma samples, which had the highest levels of cross-reactive antibody against each other, despite SSHV being more closely related to LACV. Notably, the α-SSHV plasma actually neutralized TAHV with higher titers than against homologous SSHV. These results suggest that SSHV and TAHV more closely share neutralizing epitopes with each other than they do to LACV, JCV, and INKV. From the sequence alignment of the Gc head domain, the main target for neutralizing antibodies against orthobunyaviruses^15^, there were four amino acid sites that SSHV and TAHV shared with each other that differed from the other three CSG viruses (Fig. 3, orange shading). It is possible that these positions may be in neutralizing epitope regions and therefore elicit a high cross-reactive antibody response between SSHV and TAHV, however this is just speculation as specific neutralizing epitopes have not been mapped for the CSG viruses. More likely, SSHV and TAHV have similar conformational epitopes, which are not reflected in the relationships of the primary amino acid sequences. Conformational epitopes have been implicated as important targets for the neutralization of LACV and other orthobunyaviruses^15,16,19,20^. Because of the extensive protein–protein interactions between Gn and Gc to form the tripod of Gn-Gc dimers that makes up the orthobunyavirus spike protein^14^, there are potentially many important conformational epitopes. Identifying the neutralizing epitopes will be invaluable for understanding CSG cross-neutralization and for the development of universal vaccines against these viruses.

The high degree of cross-reactivity between CSG viruses has posed an enormous challenge to the correct identification and proper diagnosis of these viruses from clinical cases. Typically, a diagnosis is confirmed by the “rule of four”, where there is at least a fourfold increase in NAb titer between acute and convalescent serum, as well as a fourfold difference in NAb titer between the suspected etiological CSG virus and other CSG viruses tested^17,18^. Even with our known α-plasma, we often could not discriminate the inoculum virus by a fourfold difference from at least one other CSG virus (Tables 3, 4, 5, 6, 7). However, with the exception of some α-SSHV plasma, the inoculum virus always had the higher ND50 (Tables 3, 4, 5, 6, 7). These results highlight the difficulty in correctly diagnosing a specific CSG virus infection, but also suggest that even in the absence of a fourfold difference, the virus with the highest NAb titer likely was the virus responsible for the infection.

Particularly concerning for correct diagnosis was the finding that some α-SSHV plasma actually neutralized TAHV, and in one case JCV, at higher titers than SSHV. It is important to note that our samples represent convalescent plasma taken four weeks post inoculation, so we do not know what the kinetics of the development of NAb against these viruses in the mice was. It is possible that evaluating NAb levels at different time points, either earlier or later post inoculation, might discriminate the inoculum virus better. These results, however, clearly indicate the need for examining several CSG viruses to try and make a proper diagnosis and suggest that non-neutralizing antibody assays such as ELISA may be an important tool, in conjunction with NAb assay results, to diagnose CSG virus infections. According to CDC arboviral diagnostic guidelines, samples are initially screened by IgM capture ELISA, and then additional PRNTs are performed to confirm a specific CSG virus diagnosis. However, due to the limited ability to discriminate CSG viruses by a fourfold neutralizing antibody titer, incorporating and/or combining ELISA antibody quantitation and neutralizing antibody titers from PRNTs may provide better diagnostic results and should be further evaluated in future work.

Additionally, using information about each virus’ geographic and mosquito vector range is critical. TAHV and SSHV primarily have discrete ranges, with SSHV in Canada and the USA and TAHV throughout Europe and Africa, but there have been reports of both in Russia^1,21^. However, the SSHV-like virus identified early on in Russia was likely the more recently characterized Chatanga virus^22^. Therefore, surveillance for the CSG viruses in mosquitoes and vertebrate hosts to better track their geographical ranges is an important consideration for diagnostics.

The level of cross-reactive antibody induced by each inoculum virus varied, with LACV, SSHV, and particularly INKV infections resulting in the highest levels of cross-reactive NAb in the mouse plasma samples (Fig. 2, Table 1). A previous study of LACV neutralization showed that synergism between multiple antibodies binding at multiples sites was critical for LACV neutralization^16^. It is possible SSHV, LACV, and INKV induce a wider range of neutralizing antibodies than TAHV and JCV, resulting in better cross-neutralization between CSG viruses. These findings may be useful for the future development of CSG virus vaccines. A previous study evaluating a CSG virus vaccine of a recombinant LACV virus expressing the M segment ORF from JCV found that the recombinant virus was attenuated in mice. This vaccine induced cross-reactive antibodies to LACV, TAHV, and JCV in monkeys, and prevented monkeys from developing viremia following JCV challenge^23^. However, in our study, JCV infection induced lower cross-neutralizing antibody compared to other CSG viruses, therefore the use of additional or different CSG virus glycoproteins in vaccine development may result in a more robust cross-reactive NAb response to the CSG viruses. Overall, these studies help to elucidate the antigenic relationships between five CSG viruses capable of causing neurological disease in humans, and may help in developing better diagnostics and vaccines for the CSG viruses.

## Materials and methods

### Viruses and cells

Virus strains LACV (human 1978), SSHV (1976), TAHV (92 Bardos), JCV (61V2235), and INKV (SW AR 83–161) were previously described^13^. Vero cells were maintained in Dulbecco modified Eagle medium supplemented with 10% fetal bovine serum (Atlas Biologics) and 1% penicillin/streptomycin solution.

### Mice and plasma

Mouse experiments were performed in full accordance with the National Institues of Health Guidelines under protocol 2016-061-E, which was approved by the Rocky Mountain Laboratories Animal Care and Use Committee (Hamilton, MT) and complies with the ARRIVE guidelines (https://arriveguidelines.org). C57BL/6 mice greater than 6 weeks of age were used and were a mix of male and female mice and were randomized for virus inoculation. Virus stocks were diluted in phosphate-buffered saline (PBS). Prior to inoculation, mice were anesthetized with isoflurane then inoculated intraperitoneally with 10^5^ PFU of each virus in a volume of 200 μl. Mice were checked twice daily following inoculation for clinical signs. None of the mice displayed clinical signs. At 29–30 days post inoculation, mice were humanely euthanized and blood collected via cardiac puncture using heparin to prevent coaggulation. Plasma was separated via centrifugation at 5000×*g* for 10 min, and separated plasma collected and stored at − 80 °C until use.

### Plaque reduction neutralization tests

Plasma collected from the mice was used in plaque reduction neutralization tests (PRNT or neutralizing antibody assay). Vero cells were plated in 24-well plates at a density of ~ 1.3 × 10^5^ cells per well. The next day, plasma samples were diluted 1:10 in DMEM supplemented with 2% FBS and 1% penicillin/streptomycin solution in a volume of 250 ul. The 1:10 dilution was subsequently serially diluted five-fold. Viruses were diluted to ~ 1 × 10^4^ PFU per ml, and 10 µl (~ 100 PFU) was added per plasma dilution. Four virus-only control samples were included per plate. The plasma dilution + virus mixtures were gently rocked by hand, then incubated at 37 °C for one hour. Media was removed from the plates of Veros, then 200 ul of the plasma dilution + virus or virus-only samples were plated per well. Plates were incubated at 37 °C for one hour to allow for virus attachment. Wells were then overlayed with 0.5 ml of MEM with 1.5% carboxymethyl cellulose (CMC), and then incubated at 37 °C. Plates with SSHV and INKV were fixed and stained at 3 dpi, and plates with LACV, TAHV, and JCV were fixed and stained at 5 dpi. Plates were fixed by filling wells with 10% formaldehyde, incubated for 1 hour, rinsed in distilled water, then stained with 0.35% crystal violet for 10–15 min, rinsed in distilled water and inverted to air dry. Plaques were counted when plates were dry.

### ND50 calculations and statistical methods

The percent neutralization was calculated for each plasma dilution by calculating the reduction in the number of plaques compared to the mean of the virus-only control wells (at least 4 wells per assay). The ND50 was calculated for each plasma sample and virus combination by plotting the percent neutralization for each plasma dilution series vs each virus and fitting an inhibition curve (inhibitor vs. normalized response) in Prism (GraphPad, Version 8.3) and calculating the inhibition concentration of 50% (IC50), which is equivalent to the ND50. The ND50s were then plotted for each plasma sample vs. each virus. Comparisons of ND50 values were done via one-way ANOVA, and for each set of anti-plasma samples Dunnett’s multiple comparisons test was run comparing the ND50s of the six anti-plasma samples between the homologous virus and each heterologous virus. Sex difference comparisons were done via unpaired t-test in Prism comparing homologous ND50 values for all viruses combined between male and female mice (males = 17, females = 13), or within each virus that had at least two of each sex (SSHV and TAHV: males = 3, females = 3; JCV: males = 4, females = 2; and INKV: males = 2, females = 4). Pearson correlations were performed in Prism between the percent amino acid identities within the Gc head domain and the heterologous virus ND50s for each inoculum virus plasma.

### Sequencing of virus stocks

RNA was isolated from stock viruses comprised of cell supernatants using the QIAamp viral RNA mini kit (Qiagen) following kit protocol with the following modifications. For lysis, 560 μl of AVL buffer without carrier RNA was used. Instead of adding 500 μl of Buffer AW1, 250 μl of Buffer AW1 was used, then an on-column DNase digest was performed using the Qiagen DNase I kit. A master mix was made with 70 μl RDD Buffer mixed with 10 μl DNase I per sample, then 80 μl of master mix was added to each sample column and incubated at room temp for 15 min. Following incubation, 250 μl of Buffer AW1 was added and tubes spun at 6000×*g* for one minute. The remaining steps were performed according to the manufacturer’s protocol. Sequencing was performed by the Research Technologies Section of the Genomics Unit at Rocky Mountain Labs, using the following protocol. One hundred nanograms of viral RNA was ribosomal RNA-depleted using Ribo-Zero Human/Mouse Rat (Illumina, San Diego CA). The depletion method was adjusted to account for lower inputs of RNA by using 16 μl of RNA, 2 μl reaction buffer and 2 μl Ribo-Zero probes for a 20 μl reaction rather than 40 μl. Ninety microliters of magnetic capture beads were washed and resuspended in 35 μl. The depleted RNA was purified with Agencourt RNAClean beads (Beckman Coulter, Brea CA) and eluted in the TruSeq Elute-Frag-Prime mix (Illumina, San Diego CA). NGS libraries were prepared following the TruSeq Total RNA-Seq protocol starting at the Elute-Frag-Prime step. The constructed libraries were assessed on a BioAnalyzer DNA1000 chip (Agilent Technologies, Santa Clara CA) and quantified using the Kapa Quantification Kit for Illumina Sequencing (Roche, Basel Switzerland). Libraries were diluted to 4 nM, pooled equally, and sequenced on the Illumina MiSeq using 11 pM as template for a Nano 300 cycle chemistry run. The sequenced reads were first trimmed and filtered for adapters and low-quality bases using Cutadapt v1.12^24^ and FastX-Toolkit^25^ respectively. Next, the cleaned reads were mapped to their respective reference genomes using Bowtie2, v2.2.9^26^, and finally, consensus sequences and variant analysis was performed using Samtools v1.8^27^. Sequence alignments of the consensus sequences for the translated Gn, Gc, and the head domain of the Gc protein were done using the ClustalW algorithm, and percent identities were calculated using MacVector (version 17.5.4).

## Data Availability

The data generated from this study are available from the corresponding author on reasonable request.
